# Building Virtual Health Training Tools for Residents: A Design Thinking Approach

**DOI:** 10.3389/fdgth.2022.861579

**Published:** 2022-06-13

**Authors:** Katharine Lawrence, James Cho, Christian Torres, Veronica Alfaro-arias

**Affiliations:** ^1^Department of Medicine, New York University (NYU) Grossman School of Medicine, New York, NY, United States; ^2^Department of Population Health, New York University (NYU) Grossman School of Medicine, New York, NY, United States; ^3^New York University (NYU) Langone Health, Medical Center Information Technology (MCIT), New York, NY, United States

**Keywords:** virtual health, telemedicine, human-centered design (HCD), user-centered design, Design Thinking, graduate medical education

## Abstract

The COVID-19 pandemic drove a rapid transition to virtual care experiences for graduate medical trainees. Core training competencies have expanded to incorporate virtual contexts, however there is limited knowledge of the optimal design of virtual care training tools for learners. In this study, we describe the application of a Design Thinking approach to the identification and co-design of novel training tools to support residents and precepting attending physicians in virtual ambulatory care practice. We applied the model of “Empathize, Define, Ideate, Prototype, and Test” *via* a mixed methods approach to (1) explore the needs, preferences, and concerns of Internal Medicine residents and outpatient precepting attendings regarding virtual ambulatory care training environments, and (2) evaluate, prototype, and test potential training tools. Eleven residents and eight attending physicians participated. Identified learner needs and problem areas included: improving virtual visit technical skills; acquiring virtual communication skills; adapting to the loss of shared in-person learning space and optimizing virtual learning environments; remediating non-virtual procedural competencies; and educating on new documentation requirements. Key solution areas included: virtual precepting support tools; digital information and education dissemination tools; and strategies for management of technical issues. Several prototypes were proposed, with a single tool (a virtual preceptor tip sheet) deployed in clinical practice. Residents found the workshop program improved their understanding of Design Thinking and its relevance to healthcare. Ultimately, Design Thinking can be deployed to engage medical trainees and precepting attendings in the effective development of novel educational tools for the virtual care learning environment.

## Introduction

The COVID-19 pandemic spurred a rapid transition to virtual healthcare models, drastically reshaping the experience of medical trainees in both graduate and undergraduate medical education (1–3). This new context of care prompted a proliferation of training initiatives aimed at preparing medical students and residents to conduct care in virtual spaces (3, 4); at the same time, major medical organizations such as the Association of American Medical Colleges (AAMC) were quick to propose telehealth competencies to guide curricular development and performance assessment (5, 6). However, even as the identification and teaching of core skills and competencies has adapted to fit virtual care environments, a knowledge gap remains regarding the optimal design, development, and use of training tools to support learners in this new context of care delivery.

As simultaneous learners and users of educational tools, medical trainees are uniquely positioned to develop innovations for the virtual health environment; often, however, their perspectives and input do not directly inform institutional models for training or care delivery. Design Thinking (DT) offers an effective framework for iteratively and collaboratively incorporating the needs, preferences, and concerns of medical trainees into the design and development of virtual health tools. Originating in computer science and popularized by design consulting and the larger field of human-centered design (7), DT employs repeating cycles of discovery, ideation, experimentation, and evolution to build tools that are appropriate and usable, thereby improving their adoption, impact, and sustainable use (8, 9). DT strategies have been utilized in a variety of different fields including business, law, and primary and higher education, as well as in healthcare delivery, quality improvement, and research (7). DT has also shown potential to aide in the development of educational strategies and programs for medical learners, although to date the majority of applications of DT to medical education have focused on curricular development (8, 10, 11). In this study, we describe the application of a Design Thinking approach to the identification and co-design of novel training tools to support residents and precepting attendings in the virtual ambulatory care learning environment.

## Methods

This study took place at a large academic medical center in New York City as it transitioned to virtual care during the COVID-19 pandemic. As part of this process, all ambulatory practices (the majority of which include physician trainees such as resident housestaff) were closed to in-person care and a rapid transition to virtual care delivery models (telephone and video visit) was undertaken. Resident trainees also transitioned to a virtual precepting experience, where review of patient care was conducted with overseeing attendings through video conference.

**Figure 1:**
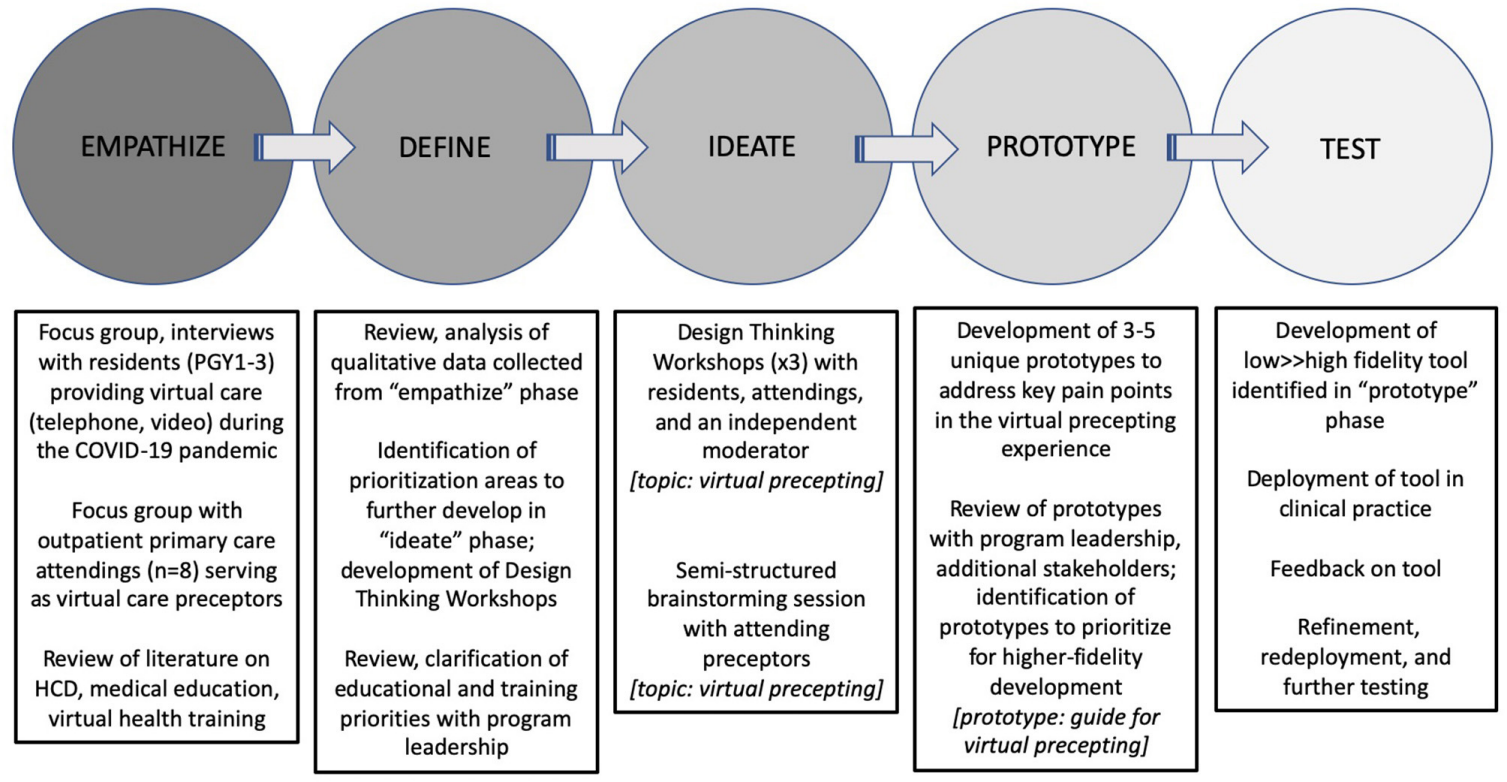
The Design Thinking process.

**Figure 2:**
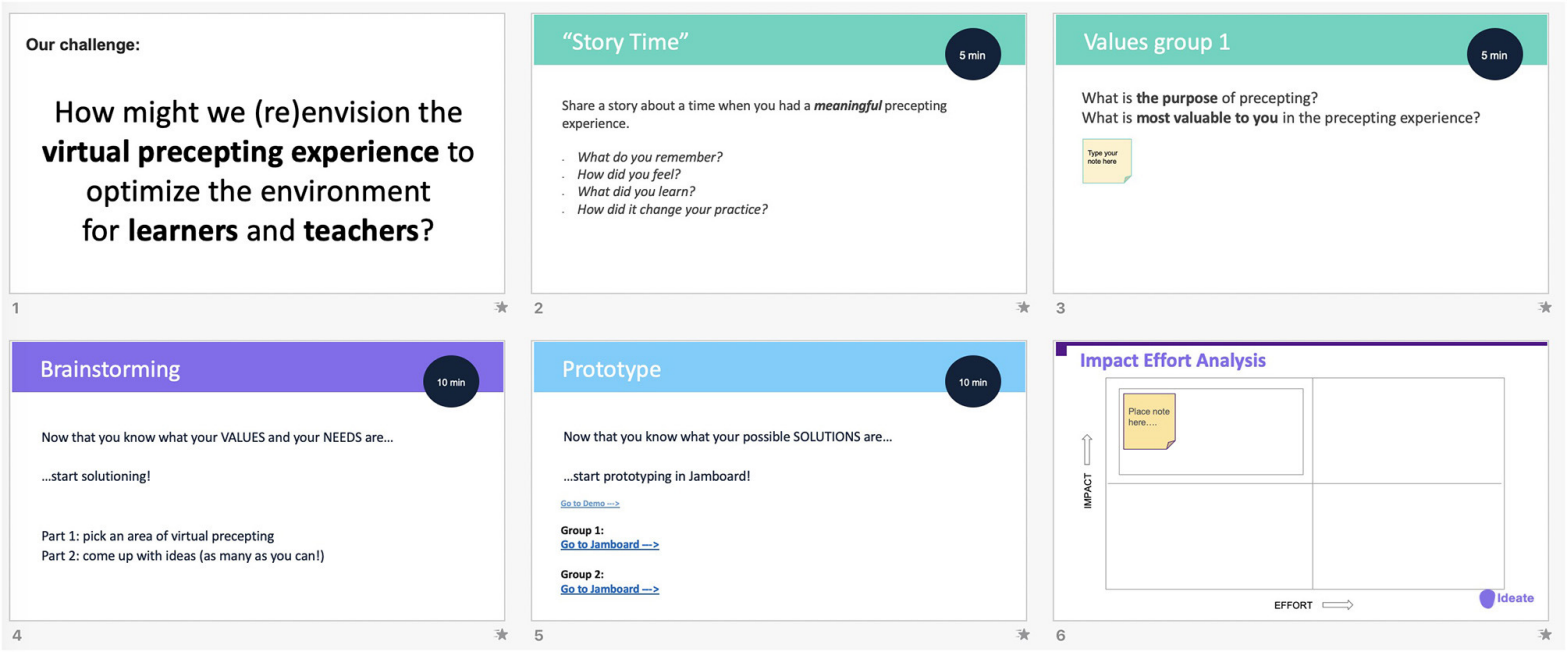
The Design Thinking Workshop.

We applied the DT model of “Empathize, Define, Ideate, Prototype, and Test” (Figure 1) to (1) explore user needs, preferences, and concerns regarding the virtual ambulatory care environment, (2) identify key problem areas and pain points for potential solutioning, and (3) brainstorm and prototype potential solutions. This model was implemented *via* a structured process consisting of three phases:

1) Exploratory interviews and focus groups (Empathize, Define): utilizing semi-structured open-ended interview questions to capture the experiences of participating residents and attendings providing virtual care during the pandemic, and to identify key needs, preferences, and concerns regarding the current virtual care environments. Qualitative results of this phase were analyzed for major and minor themes, with the goal of identifying topic areas for further structured exploration, problem identification, and solutioning.
2) Design Thinking Workshop (Ideate): tailored to a specific scenario identified from the preceding qualitative work, the DT Workshop utilizes the “Empathize, Define, Ideate, Prototype, and Test” structure to guide participates through a series of exercises to creatively engage with a problem and brainstorm potential solutions, with the goal of identify a single solution considered optimal for actual development (Figure 2). Along with the Workshop, an 8-question 3-point Likert Scale (disagree, agree, neutral) survey to assess key training objectives was administered to residents post-Workshop *via* RedCap, a secure web application for building and managing online surveys (Table 1) (12).
3) Rapid prototyping (Prototype, Test): before proceeding to development, potential solutions identified from the Workshops were evaluated for feasibility by the research team and key stakeholders using an “impact/effort matrix”, a tool derived from Lean Six Sigma methodologies to rapidly evaluate and prioritize projects (Figure 2) (13). Selected solutions were then developed into a minimum viable product (MVP) for deployment into clinical practice, with the goal of soliciting feedback from users regarding its features and recommendations for further development and refinement.

**Table 1:** Workshop post training survey questions.

1) I have a better understanding of Design Thinking
2) I understand how Design Thinking can be applied to challenges in healthcare
3) Design Thinking can help solve technical challenges in healthcare
4) Design Thinking is relevant to my clinical practice
5) Design Thinking is relevant to my professional development
6) Design Thinking is relevant to my research
7) Please provide your OVERALL rating of the quality of this training
8) Would you recommend this training to others?

## Results

Eleven Internal Medicine residents and eight outpatient precepting attendings participated in this study. Nine residents (81%) responded to the post-Workshop online survey. All nine (100%) indicated improved understanding of Design Thinking and its relevance to healthcare. The majority “agreed” that Design Thinking can solve technical challenges in healthcare (*n* = 7, 78%) and with the relevance of Design Thinking to their clinical practice (*n* = 5, 56%), and “neutral” on the relevance to their professional development and research (*n* = 5, 55 and 7, 78%, respectively). The overall program was rated “good” to “very good”, with the majority (*n* = 6, 67%) recommending the program to others.

Major learning from each phase of the iterative Design Thinking process included:

**Empathize**: During the qualitative interviews with residents and attendings, learners reflected on their experience with the telemedicine transition in their clinics (“It felt like the whole purpose of the visits was different. [W]e were doing more triaging, trying to determine if patients needed to come to the hospital…that's a very different skill”) and its impact on their training, particularly the loss of the shared in-person learning environment of the precepting offices (“I miss hearing my colleagues present their cases [in the shared precepting room]. I feel like I learned so much from those encounters…that is lost now”). Attendings reflected on both the learning opportunities (“although there is information missing from the patient-care encounter [e.g., vital signs and exam] I still feel like there is plenty to teach the residents”) and challenges (“a lot of ‘teaching' during precepting has gone to workflow issues, and I feel like I'm not doing enough to teach and support the residents”). Key areas of need for the resident learner were identified, including: increasing skill and comfort with technical processes and workflows of the virtual visit platform; adapting skills for virtual communication and rapport-building with patients; improving the current virtual precepting environment; remediating training competencies not addressed through virtual experiences (e.g., procedural skills); and incorporating new virtual visit documentation requirements.

**Define**: The themes identified in the “Empathize” phase were reviewed with a panel of key stakeholders including residents, attending physicians who provided precepting to residents, program leadership, and content experts in medical education, to identify topic areas for further exploration and solutioning in a Design Thinking Workshop. Potential topics were evaluated using the impact/effort matrix to identify a single topic with the highest likelihood for successful solutioning and implementation based on factors including perceived educational value, cost, time, institutional support/enthusiasm, and available technical resources (e.g., EHR analysts, developers) and constraints. The virtual precepting experience was identified through the matrix, with the problem statement “*How might we (re)envision the virtual precepting experience to optimize the environment for learners and teachers?*” created to guide the development of the workshop.

**Figure 3:**
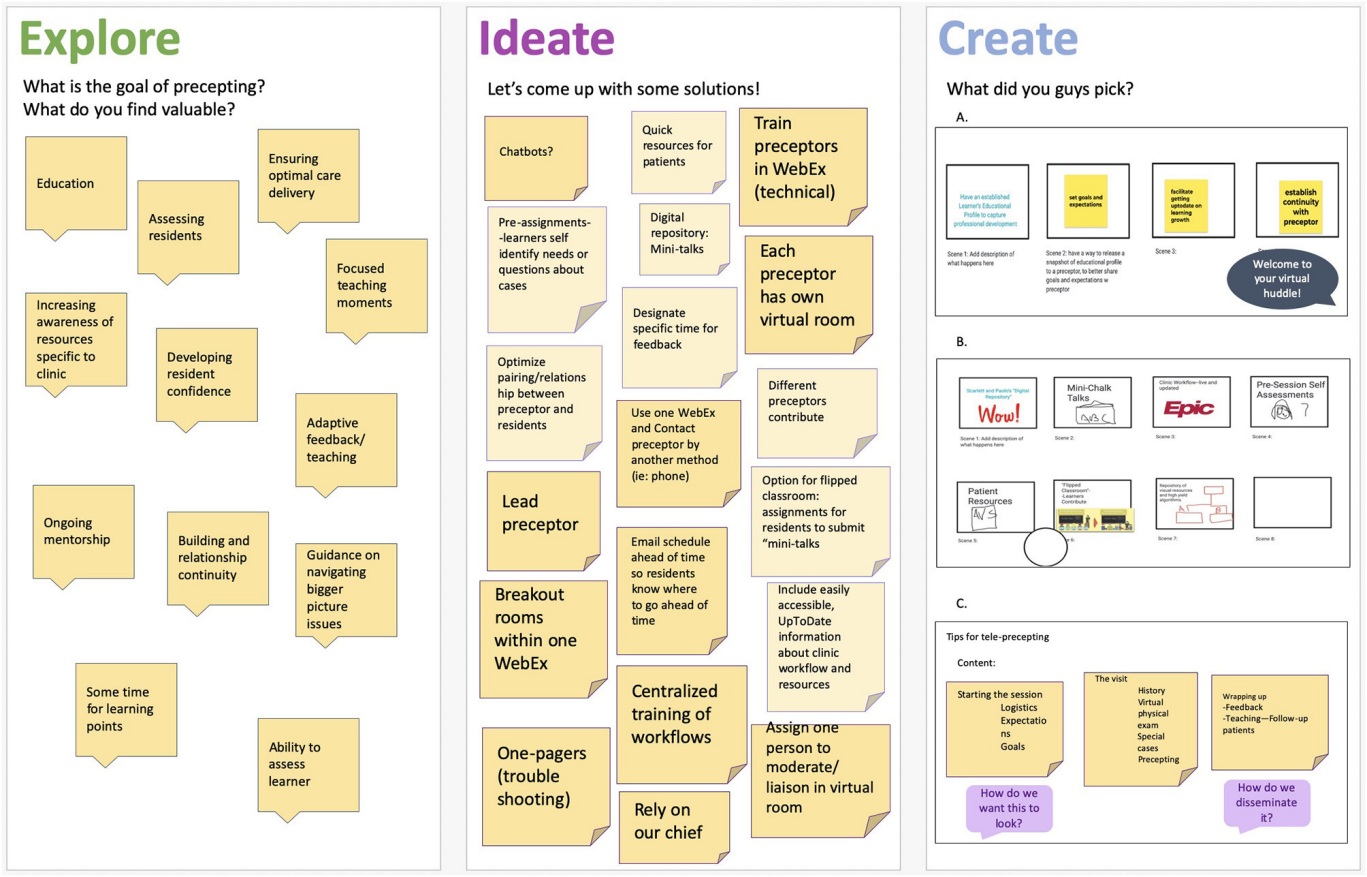
Content and prototypes from the combined Design Thinking Workshops.

**Ideate**: Three virtual workshops were conducted (2 with residents, 1 with attendings). Using a three-phase interactive sequence (Explore, Ideate, and Create), participants were divided into 2-or 3-person virtual breakout groups and asked to (1) identify a key problem in current virtual precepting, (2) brainstorm possible solutions, and (3) design and present a low-fidelity prototype of one solution (Figure 3). In the “Explore” phase, key problems identified in the current virtual precepting experience included: disjointed communication practices between preceptors and trainees (e.g., calling preceptors over phone vs. video conference); the lack of structured goal and expectation setting for the precepting sessions; the absence of structured virtual information dissemination practices (e.g., clinic updates); the loss of shared physical learning space with colleagues; and ongoing challenges with the management of technical issues. In the “Ideate” phase, participants collaboratively developed over 30 solutions to identified problems. In the “Create” phase, proposed solutions selected by participants for feasible development included:

- A tip sheet for virtual preceptors: a concise, easy-to-access tool for use by attending preceptors to structure “virtual precepting sessions” (both for virtual patient visits *and* remote precepting), which would highlight high-yield areas to enhance trainee learning, promote rapport, and improve efficiency and effectiveness of learners' clinical time.
- Digital shared-learning plans for residents and preceptors: a digitally available, dynamic shared portfolio of self-identified learner goals for residents to achieve during clinical experiences. This tool would be shared with the corresponding preceptor in advance of a precepting session, to allow preceptors to tailor teaching content to goals of the resident. Future iterations of this “digital portfolio” would move with residents throughout their in- and out-patient experiences, and longitudinally through their training, and align with major competencies and training goals of the institution.
- A real-time virtual clinical bulletin board: in response to continual changes to clinic resources, this digitally-available tool would be shared by preceptors at the beginning of each virtual precepting session, highlighting up-to-date practice information (e.g., contact information of front desk staff, availability of specialty services such as physical and occupational therapy, how to initiate e-consults) and other relevant updates for clinic residents. Information would be updated and maintained by practice managers.
- An integrated virtual team huddle: prior to the pandemic, clinical staff conducted daily interdisciplinary team “huddles” prior to each clinic session, with the purpose of sharing practice updates, clarifying team roles, and building team rapport. This event would be transitioned to a virtual platform (e.g., Zoom) and conducted prior to the clinical session, so that learners could better identify and utilize their clinical support staff, make appropriate referrals, and improve care connections and follow-up for their patents.
- “Just-in-time” digital chalk talks: leveraging digital resources such as Up-To-Date and NEJM Knowledge+ as well as the novel virtual precepting environment, precepting attendees would create succinct case-based learning activities for residents as part of their precepting session. These “digital chalk talks” would be made available as part of an open-source knowledge repository that preceptors could access in real-time throughout a precepting session, and tailor to the cases being seen by learners.

**Prototype and Test**: Proposed solutions from the Workshops were reviewed with program leadership and content experts, who used an additional impact/effort matrix to identify the tip sheet for virtual preceptors as the highest yield prototype to further develop.

Content for the tool was co-developed between the researchers and study participants, and reviewed by medical educators and attending physicians for feedback and refinement. Consensus was on a concise, 1–2 page document that could be made easily available to precepting attendings, with the goal of helping them structure and facilitate an effective remote precepting session. Areas of focus included: starting the precepting the session, case precepting specifics, and session wrap up. Tip content was developed and refined through feedback from participating residents, attending-users, and medical education content experts. Tip domains for precepting and teaching included: sharing logistics and setting expectations for each precepting session; building “virtual rapport” with learners; teaching how to take advantage of the virtual environment to explore new areas of patient care (e.g., “virtual home visits”, home medication review); teaching specific virtual competencies (e.g., virtual physical exam skills); fostering the virtual patient-provider relationship; planning for patient follow-up; and closing a session by sharing case-based learning points and providing feedback. This tool was ultimately deployed among attendings in the primary clinic as a paper-based PDF document, as well as shared widely for clinical use and feedback through the AAMC's Clinical Teaching and Learning Experiences curated collection (Appendix 1).

## Discussion

Using a Design Thinking approach among residents and attending preceptors engaged in virtual care delivery training, this study identified (1) the experiences, needs, preferences, and concerns of medical learners who engage in virtual care as part of their training, (2) key problem areas in the current virtual health training environment, and (3) multiple potential solutions and specific prototypes to develop, deploy, and evaluate in practice. Through the DT process, the research team worked alongside key stakeholders at each step to co-design, refine, and deploy an optimized intervention to address a critical gap in the current training experience—that of the virtual precepting experience. In the assessment of resident participants, learners found the workshop program improved their understanding of DT and its relevance to healthcare, including to addressing technical challenges in healthcare systems and their own clinical practice, although the impact of the participatory workshop experience on their personal research and professional development was less clear.

This research's contribution is in the successful application of DT to address problems of an emerging care technology in real time as they are identified by the end-users of that technology (e.g., residents and supervising attendings). To our knowledge, this is one of the first studies to use DT strategies for the co-design and development of just-in-time pragmatic tools to support medical learners in the virtual care environment. The majority of work examining the use DT in medical education has focused on either the explicit teaching of DT to medical trainees or the incorporation of DT philosophy into the development of medical curriculum more broadly. In a comprehensive article by social science scholar Madson (14) that provides a concise overview of how design thinking has been understood and operationalized in medical education, the author notes that most DT initiatives in medical education are in the early stages, and take the form of programs, courses, workshops, and hackathons. In a similar qualitative review of DT frameworks in health professions education (including nursing, pharmacy, physiotherapy, occupational therapy, art, and engineering) McLaughlin et al. (10) identified 15 studies evaluating the use of DT in educational programming; they found most studies used Design Thinking as a methodology to produce and examine other outcomes such as self-efficacy and confidence, participant experiences, program characteristics, or solutions to a specific problem. In the field of healthcare technology and innovation (although not specifically virtual health), Carter et al. (15) discuss the application of human-centered design principles to the design of curriculum to foster clinician-innovators; however, their focus is on recommending strategies for medical educators and institutions to ensure they are incorporating healthcare innovation into their offerings, rather than applying DT processes to specific educational products. In the larger world of telemedicine commercial product development and practice, considerable work has been done to apply insights from human-computer interface (HCI) and human-centered design to the design, technical development and integration, and user experience of telemedicine platforms and tools; however, it is unclear that medical learners have been meaningfully incorporated in this work, or that medical training *via* these tools is considered a priority. As of completion of this manuscript, a search of the use of DT in the virtual health or telemedicine *training* returned no articles specific to this topic; one article, by Thakur et al., (16) does briefly review the expansion of telehealth as one of a number of disruptions to medical education caused by the COVID-19 pandemic, and suggests that DT principles and strategies can be used to solve emergent complex problems, including those related to training as a result of the pandemic.

Limitations of this study include generalizability due to the small sample size within a single training environment experiencing a particular instance of telehealth (e.g., rapid expansion during a global pandemic); however, a core feature of the Design Thinking process is its emphasis on local contexts (including stakeholders) in order to create specifically tailored, usable products—therefore, generalizability may not be a desired outcome. We also identified constraints at the program and institutional level to effectively incorporating, testing, and sustaining recommendations around virtual health training innovations; while several potentially effective solutions to virtual health training challenges in our system were identified, resource and other constraints prevented their further exploration and implementation. Finally, given the scope and time limitations of the study, we were not able to assess the long-term impact of our training tool; further research is needed to better understand the diffusion of this novel educational tool into practice, particularly its sustained adoption, adaptations, and/or abandonment. In general, more research is needed to understand future use cases of virtual healthcare technology in medical education, and their effects on the trainee experience.

## Conclusions

Design Thinking can be deployed to engage medical trainees and their supervising attendings as users in the creative ideation, solutioning, and co-design of novel educational tools for the virtual care environment, potentially improving their acceptability and use. As Design Thinking becomes more fully integrated into medical education—including not only the teaching of DT skills to learners but the active use of DT strategies to develop effective learning tools, products, and strategies—efforts should be undertaken to ensure that learners are incorporated as key stakeholders in curricular design and learning tool development, particularly in the areas of emerging health technologies such as virtual health.

## Data Availability Statement

The raw data supporting the conclusions of this article will be made available by the authors, without undue reservation.

## Author Contributions

KL was responsible for the design of the study, oversight of Design Thinking Workshops, and manuscript writing and preparation. JC was responsible for the design of the study, workgroup discussions, and manuscript writing and preparation. CT was responsible for the workgroup discussions and manuscript writing and preparation. VA-a was responsible for developing and conducting the Design Thinking Workshops and manuscript writing and preparation. All authors contributed to the article and approved the submitted version.

## Funding

This research was supported by a grant from the NYU Grossman School of Medicine Program for Medical Education Innovations and Research (PrMEIR), which provides funding for the exploration of innovations in medical education and training.

## Conflict of Interest

The authors declare that the research was conducted in the absence of any commercial or financial relationships that could be construed as a potential conflict of interest.

## Publisher's Note

All claims expressed in this article are solely those of the authors and do not necessarily represent those of their affiliated organizations, or those of the publisher, the editors and the reviewers. Any product that may be evaluated in this article, or claim that may be made by its manufacturer, is not guaranteed or endorsed by the publisher.
