# Supplementary material for: Building Virtual Health Training Tools for Residents: A Design Thinking Approach
Source: Front Digit Health. 2022 Jun 13;4:861579. doi: 10.3389/fdgth.2022.861579 (PMC9234169; doi:10.3389/fdgth.2022.861579)
Supplement: Supplementary file 1 [file Table_1.DOCX]

**Tips for Telepreceptors**

While much of the virtual precepting environment can be built to mimic the traditional “in-clinic” experience for trainees and preceptors, virtual precepting offers unique challenges and opportunities to engage with, educate, and assess the competencies and needs of learners. Developing a systematic approach to virtual precepting can help preceptors optimize clinical care, maximize learning, and foster the trainee-preceptor relationship.

This document provides guidelines, tips, and recommendations for the remote preceptor working with residents who are providing telemedicine ambulatory care.

| **Starting the Session**  Have a virtual huddle between the preceptor, trainees, and other team members (nurses, administrative staff, medical students) to make sure everyone is present and on the same page. | |
| --- | --- |
| **Sharing Logistics** | - Provide updates and make announcements about any recent changes to clinic flow, staffing, or resources - Discuss how you want to precept cases (e.g. one vs. multiple cases at a time,) - Discuss how you want to share learning points with the group (e.g. via chat, shared document, etc.) - Have a plan to address technical failures and/or workflow interruptions that may occur during the session   - Make sure trainees are familiar with and have easy access to alternative technologies to reach patients   - Share contact numbers in case of internet connectivity issues |
| **Setting Expectations** | - Ask your trainees to always present on camera - Establish a clear expectation of how the trainee will communicate the finalized assessment and plan to the patient after precepting - Consider giving an assignment for the session, such as a focus on health maintenance, assessment of social determinants of health like food insecurity, or topics identified by the learner - Set a time for end-of-session huddle (*see below*) |
| **Building Rapport with Learners** | - Include personal check-ins with learners - Allow time for a group discussion (including social discussions) among team members and learners - If trainees are at home, ask them to share something in their work area that has meaning (e.g. show their nearby art, bring their pets on camera, etc.) |

| **Precepting a Case**  The format can be very much the same as in-person precepting, but with new opportunities in the virtual environment. | |
| --- | --- |
| **Taking Advantage of the Virtual Environment** | - Sharing the Screen   - Consider having the trainee share their screen so they can show you labs and other information from the EHR as they present (with proper attention to PHI security)     - This also helps avoid potential distractions from each person looking at their own EHR screen   - Other than the EHR, you can screen share:     - Quick literature searches to answer your clinical questions     - Group shared documents, clinic documents, other practical info - Linking Back to Learning   - Use the chat box, a shared document, or other space for:     - A running list of helpful links, relevant journal articles, and other resources     - Clinical pearls from individual cases     - Asking and answering questions |
| **Exploring New Areas of Patient Care** | - Expanding the Social History   - Take opportunities to teach learners to ask additional relevant questions regarding social background and/or needs (e.g. home visits, social or occupational exposures, screening for social isolation etc.)   - Explore digital determinants of health, such as internet access and quality, financial burdens, and resources - Planning for Health Maintenance   - Discuss with learners how to prioritize routine health maintenance such as vaccines and cancer screening during times of disrupted or limited care, and to plan with patients how to obtain needed services |
| **Teaching Virtual Competencies** | - Consider incorporating short telemedicine-specific teaching into either individual cases or structured teaching with trainees (e.g. virtual physical exam tips, information gathering from virtual home visits etc.) |
| **Fostering the Patient-Provider Relationship** | - While virtual care provision can feel disruptive to building relationships between trainees and their patients, there are also opportunities to work longitudinally with patients, including communicating asynchronously with patients and scheduling mixed in-person and telemedicine visits |

| **Closing the Session**  It can be easy to let the session end with the last precepted case, but there is plenty of learning to be had in one final team huddle or discussion. | |
| --- | --- |
| **Planning for Follow-up** | - Discuss plans for follow-up of labs, imaging studies, and other orders - Decide on specific plans for complex cases that may require between-visit care or coordination |
| **Sharing Learning Points** | - Learners can share brief learning points from individual cases or newly discovered tips on workflow |
| **Providing Feedback** | - Collect feedback on visit flow, logistics, and other areas; discuss areas for improvement in future tele-precepting sessions - Give at least one point of feedback to each trainee on their case presentations/management - Set the expectation (for yourself) to give feedback on trainees’ notes from the session |

Developed by Katharine Lawrence, James Cho, and Christian Torres at NYU Grossman School of Medicine and Bellevue Hospital Center. Support for this project provided by the NYU PrMEIR Program.
